# Multi-Objective Optimization of Fused Deposition Modeling for Mechanical Properties of Biopolymer Parts Using the Grey-Taguchi Method

**DOI:** 10.1186/s10033-023-00847-z

**Published:** 2023-02-27

**Authors:** Kapil Kumar, Hari Singh

**Affiliations:** 1 Department of Mechanical Engineering, National Institute of Technology, Kurukshetra, 136119 India; 2 Department of Mechanical Engineering, National Institute of Technology, Kurukshetra, 136119 India

**Keywords:** Fused deposition modeling, Mechanical properties, Taguchi method, ANOVA, Grey relational analysis, SEM

## Abstract

The urgent need to develop customized functional products only possible by 3D printing had realized when faced with the unavailability of medical devices like surgical instruments during the coronavirus-19 disease and the on-demand necessity to perform surgery during space missions. Biopolymers have recently been the most appropriate option for fabricating surgical instruments via 3D printing in terms of cheaper and faster processing. Among all 3D printing techniques, fused deposition modelling (FDM) is a low-cost and more rapid printing technique. This article proposes the fabrication of surgical instruments, namely, forceps and hemostat using the fused deposition modeling (FDM) process. Excellent mechanical properties are the only indicator to judge the quality of the functional parts. The mechanical properties of FDM-processed parts depend on various process parameters. These parameters are layer height, infill pattern, top/bottom pattern, number of top/bottom layers, infill density, flow, number of shells, printing temperature, build plate temperature, printing speed, and fan speed. Tensile strength and modulus of elasticity are chosen as evaluation indexes to ascertain the mechanical properties of polylactic acid (PLA) parts printed by FDM. The experiments have performed through Taguchi's L_27_ orthogonal array (OA). Variance analysis (ANOVA) ascertains the significance of the process parameters and their percent contributions to the evaluation indexes. Finally, as a multi-objective optimization technique, grey relational analysis (GRA) obtains an optimal set of FDM process parameters to fabricate the best parts with comprehensive mechanical properties. Scanning electron microscopy (SEM) examines the types of defects and strong bonding between rasters. The proposed research ensures the successful fabrication of functional surgical tools with substantial ultimate tensile strength (42.6 MPa) and modulus of elasticity (3274 MPa).

## Introduction

Additive manufacturing (AM), alternatively known as 3D printing (3DP), is continuously proving itself as a revolutionizing technique due to a remarkable reduction in product development cycle time in designing and rapid manufacturing of a new product. The AM can build even a complex part with a high level of customization in a single step without requiring specific material handling equipment. Due to this, it is an attractive technology for modern-day applications, including biomedical, engineering, intricate objects, automotive components, and green manufacturing of parts.

In the fused deposition modelling (FDM) process, the 3D object builds additively from the bottom to the top layer in the exact shape of the CAD model. The 3D geometrical CAD model of an object converts into STL format, then uploaded into apposite software, where the STL file slices in layers before sending it to the 3D printer machine. The FDM observes striking a balance between cost-effectiveness and strength of parts for a given thermoplastic polymer; therefore, it is pretty popular among novice researchers for quick prototyping and manufacturing functional parts [1, 2].

## Literature Review

Several studies explored the significant process parameters largely accountable for enhancing the mechanical properties of FDM printed parts. Gordon et al. [3] implemented Taguchi orthogonal L_8_ array and analysis of variance (ANOVA) to investigate the influence of various process parameters on tensile, fracture strengths, and critical stress intensity factor of FDM printed polylactic acid (PLA) components. They used process parameters such as layer thickness, relative density or infill percentage, extrusion temperature, speed, infill direction, and perimeter layer (or outer shell). Irrespective of orientation, a high value of infill percentage (relative density) is the most influential setting for enhancing the samples' tensile strength and fracture strength. Chacon et al. [4] analyzed the printing parameters, namely, build orientation, layer thickness, and feed rate, for enhancing the mechanical properties of the FDM parts, such as tensile strength, flexural strength, ductility, and stiffness. It concludes that the upright orientation results in the lowest mechanical properties, while on-edge and flat orientations give the highest strength and stiffness. The mechanical properties increase with increasing layer thickness and decrease with increasing feed rate for the upright orientation. Yao et al. [5] established a theoretical model based on the transverse isotropic hypothesis, classical lamination theory, and Hill-Tsai anisotropic yield criterion to estimate the ultimate tensile strength of PLA materials printed by FDM in seven different angles (0°, 15°, 30°, 45°, 60°, 75°, 90°) with three-layer thicknesses (0.1 mm, 0.2 mm, 0.3 mm) for each angle. It experimentally observes that tensile strength increases with a decrease in layer thickness from 0.3 mm to 0.1 mm for the same print angle. Sood et al. [6] studied the effects of layer thickness, orientation, raster angle, raster width, and air gap on improving the mechanical properties such as tensile, flexural, and impact strength of ABS parts printed by FDM. They developed the mathematical model by central composite design (CCD) and analyzed it by ANOVA. They observed that tensile strength increased with increasing the layer thickness, higher value of orientation, increasing the raster angle, and small air gap. A small raster angle increases the stress accumulation and distortion but results in more strength of the built parts. Thick rasters increase the stress accumulation along the width of the part resulting in high temperatures near the boding surfaces, which enhances the diffusion and strong bond formation. Flexural strength decreases with increasing layer thickness at a constant value of raster width. Wu et al. [7] studied the influence of process variables on mechanical properties like tensile strength, bending strength, and compressive strength of FDM-printed polyether-ether-ketone (PEEK) samples. Three levels of layer thickness and three raster angles were considered process variables of FDM. A layer thickness of 0.3 mm and a raster angle of 0° were observed as optimal process variables to obtain optimal mechanical properties. Lanzotti et al. [8] developed a second-order response surface model to establish a relationship among process parameters of the FDM process. These process parameters were layer thickness, infill orientation, number of shell perimeters, and the response variables such as tensile strength, elastic modulus, and strain at failure of PLA parts. It observed that ultimate tensile strength (UTS) increases with increasing values of the layer thickness and number of perimeters and decreasing values of the infill orientation. The smaller layer thickness, higher value of shell perimeters, and lower value of infill orientation result in the highest elastic modulus value. Attoye et al. [9] used the design of experiments (DOE) to optimize different process parameters, i.e., nozzle temperature, printing speed, and print orientation, to ascertain the best values of Young's modulus and yield strength and ultimate strength of FDM manufactured parts. Le et al. [10] explored the quantitative relationship between process parameters, i.e., layer thickness, deposition velocity, and infill rate, and tensile strength of FDM-built parts made of PLA. The results exhibited that tensile strength and bonding degree decrease continuously with increasing layer thickness values from 0.05 mm to 0.35 mm. The same trend was followed by the tensile strength and bonding degree while increasing the deposition velocity from 30 mm/s to 100 mm/s. These properties continuously increased, increasing the infill rate from 50% to 100%. Liu et al. [11] proposed an experimental research approach based on the Taguchi method (L_27_) and ANOVA to investigate the influence of printing parameters. These parameters were deposition orientation, layer thickness, deposition style, raster width, and raster gap on three evaluation indexes of mechanical properties, such as tensile strength, flexural strength, and impact strength of FDM-built parts made of PLA. Finally, Grey relational analysis searches out a combination of optimal process parameters to optimize comprehensive mechanical properties. It concluded that the deposition rate is the most significant factor, followed by layer thickness and deposition style. Drummer et al. [12] investigated the influence of different nozzle temperatures to evaluate the tensile strength of FDM printed parts made of biodegradable PLA. Increasing the nozzle temperature produces a high degree of crystallinity. Khatwani et al. [13] experimentally investigated the influence of process parameters such as nozzle diameter, layer thickness, and part bed temperature on mechanical properties, namely, tensile strength and flexural strength of FDM parts made of PLA. Increasing part bed temperature results in increasing tensile strength and flexural strength. However, tensile strength decreases and flexural strength increases with gradually increasing layer thickness. Akhoundi and Behravesh [14] extensively investigated the effect of filling pattern and infill percentage on tensile strength, flexural strength, and modulus of FDM printed parts made of PLA. Nugroho et al. [15] investigated the effect of the different layer thicknesses of PLA parts. The results revealed that higher thickness layers yield the parts of higher strength with lower ductility. Zhou et al. [16] studied the effect of the printing pattern and infill density on the ultimate tensile strength (UTS)/weight ratio and the modulus of elasticity of FDM printed PLA parts by using Taguchi L9 orthogonal array. The experimental results indicated that smaller air gaps and triangular infill patterns were beneficial for obtaining a good UTS/weight ratio. For the same cross-sectional area, the portion of solid strands to the air gap increases with an increase in infill density resulting in increased tensile strength. Garg et al. [17] prepared hip replacement implants using an ABS plastic pattern through investment casting. The pattern and implants were optimized to achieve the best surface finish, hardness, and dimensional accuracy by adjusting the process parameters of the FDM process. Taguchi's L18 orthogonal array (OA) was employed to reveal the influence of six process parameters such as type of pattern, V/A ratio of the casting, the orientation of the pattern in the FDM machine, the density of the pattern, mould thickness, and grade of material. ANOVA was applied to find the optimal process parameters for optimizing individual responses. Subsequently, multi-objective optimization results in the implants’ best surface finish, hardness, and dimensional accuracy. Srivastava et al. [18] performed a multi-objective optimization for responses, namely, built time and support volume in input process parameters such as slice height, contour width, air gap, raster width, raster angle, orientation by using central composite response surface methodology (RSM) design coupled with fuzzy-logic. RSM designs the experiments on the FDM printer with ABS as a filament. The RSM-based fuzzy logic, a multi-objective optimization tool, was successfully implemented for the FDM process. Equbal et al. [19] performed the multi-objective optimization through the weighted principal component analysis (WPCA)-based desirability function method to obtain the best optimal combination of input process parameters of FDM. These process parameters were layer thickness, air gap, and raster angle for minimizing the relative changes in output characteristics like length, width, and thickness. The RSM-based central composite design performs the experimentation on acrylonitrile butadiene styrene (ABS) polymer. The multi-objective optimization concluded that desired multi-outputs could achieve by the optimal combination of raster width, air gap, and raster angle as 0.4064 mm, − 0.004 mm, and 30°, respectively. Nguyen et al. [20] performed multi-objective optimization using the Taguchi method coupled with non-dominated sorting genetic algorithm II. The input process parameters, like layer height, infill percentage, printing temperature, printing speed and weight, tensile strength, and printing time as multi-outputs used to develop mathematical models for the FDM process using PLA material.

It concluded that the Taguchi method, coupled with the non-dominated sorting genetic algorithm II, can be successfully used in practice. Shilpesh et al. [21] have investigated the effect of process parameters, such as raster angle, layer height, and raster width, on the tensile strength of FDM-printed PLA parts. The results indicate the highest tensile strength obtained at 0° raster angle with the lower layer height value. PLA has excellent tensile and flexural strength and may derive from renewable resources such as carbon dioxide, wheat, corn, and rice. PLA is environment-friendly, recyclable, compostable, biodegradable, biocompatible, and non-toxic. It also has no reported carcinogenic effects. So, food and drug administration (FDA) approved the PLA for direct contact with biological fluids. PLA has also proven to maintain robust sterility, which helps to mitigate infection [22]. The melting point of PLA is relatively low (150−160 °C); hence, relatively less energy is required to print the material [23]. Among all the polymers, PLA is one of the most prominently chosen filament materials for the FDM 3D printing process.

It is evident from the literature review that various researchers have studied the mechanical properties of the FDM printed parts by considering the influence of a limited number of process parameters. There is thus an enormous scope to thoroughly investigate the mechanical properties of the biopolymer PLA parts printed via the FDM process under the consideration of process parameters as more as possible. Several researchers have performed multi-objective optimization with principal component analysis (PCA), WPCA-based desirability function, RSM-based fuzzy-logic, Taguchi method coupled with non-dominated sorting genetic algorithm II, etc. In the current scenario, Taguchi-based grey-relational analysis has also gained substantial importance because of transforming multi-responses into a single function that is, of course, easy to handle. In actual practice, the fabricated part must possess more than one mechanical property concurrently to overcome various loadings. Therefore, it is necessary to trade off various mechanical properties in a fabricated part per the customers' demand. Grey relational analysis has been employed for multi-response optimization of quality characteristics to address this concern.

## Experimental Plan

### Selection of Process Parameters and Their Levels

This paper mainly explores the influence of process parameters on the mechanical properties of the FDM printed parts. These parameters are layer height (A), infill pattern (B), top/bottom pattern (C), number of top/bottom layers (D), infill density (E), flow (F), number of shells (G), printing temperature (H), build plate temperature (I), printing speed (J), and fan speed (K). All discussed process parameters in the literature survey are available in the latest slicer software Ultimaker Cura 4.9.1. In order to identify the feasible range of process parameters performing screening experimentation that follows a one-factor-at-a-time approach. The levels of the printing parameters were selected as given in Table 1.

**Table 1:** Levels of process parameters

Process parameters	Units	Levels		
		I	II	III
A	mm	0.1	0.2	0.3
B	−	Grid	Triangle	Concentric
C	−	Line	Concentric	Zig-zag
D	−	2	3	4
E	%	50	75	100
F	%	100	125	150
G	−	2	3	4
H	℃	200	210	220
I	℃	50	55	60
J	mm/s	40	60	80
K	%	10	30	50

### Experimental Procedure

**Figure 1:**
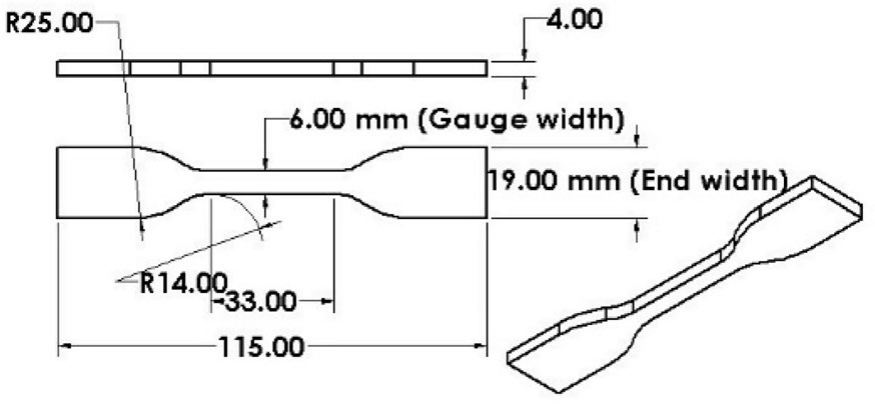
Tensile specimen dimensions (mm)

**Figure 2:**
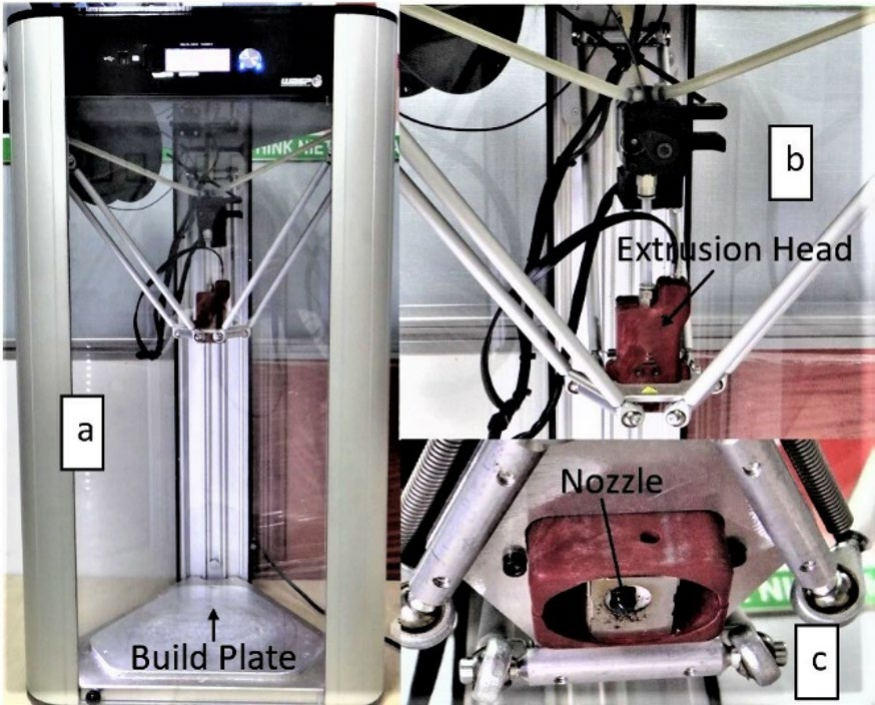
**a** 3D printer, **b** close up view of extrusion head, **c** close-up view of the nozzle

ASTM D638 16 standard considered to design the 3D model of the tensile test specimen in the solid-work software. Figure 1 indicates the schematic diagram and dimensions of the specimen. A common desktop printer, Delta Wasp 2040 Turbo2, is used for rapid manufacturing and prototyping of small-scale functional products, as shown in Figure 2. The printer is a single extruder having a nozzle diameter of 0.4 mm, which uses a filament of 1.75 mm diameter. This study considered 11 control parameters, and their values were adjusted by open-source slicer software, Ultimaker Cura 4.9.1. The experiments are designed based on Taguchi's DOE approach. Here, 11 process parameters, each with three levels, are considered, which have 22 degrees of freedom (DOFs). Therefore, Taguchi's L_27_ (3^13^) OA has 26 DOFs specifically chosen for the experimentation. Many factors under consideration eliminated this article's scope of the interaction study. A flat position (*X-Y* plane) in which the deposition of the fused filament aligns in the direction of pulling was selected to print the samples through 3D printer. Each experiment is replicated three times for printing tensile test specimens. The experimental raw data of testing and signal-to-noise (S/N) ratio data for all responses indicate in Table 2. A direct contact micro-computer-controlled electronic horizontal extensometer (Model PC-2000, capacity 20 kN) measured the tensile stress and drew the stress-strain diagrams of each test with a crosshead speed of 3 mm/s. The slope of the stress-strain diagram determines the modulus of elasticity (E).

**Table 2:** Experimental raw data and S/N ratio data of both responses based on L_27_ OA

Exp No.	A	B	C	D	E	F	G	H	I	J	K	UTS (MPa)				E (MPa)			
												Trial 1	Trial 2	Trial 3	S/N ratio (dB)	Trial 1	Trial 2	Trial 3	S/N ratio (dB)
1	1	1	1	1	1	1	1	1	1	1	1	19	17	19	25.23	2221	2234	2265	67.00
2	1	1	1	1	2	2	2	2	2	2	2	24.7	25	24	27.78	601	614	645	55.84
3	1	1	1	1	3	3	3	3	3	3	3	28.6	29	30	29.31	654	667	698	56.55
4	1	2	2	2	1	1	1	2	2	2	3	17.7	18	17	24.86	960	975	999	59.80
5	1	2	2	2	2	2	2	3	3	3	1	25.2	26	26	28.13	548	561	592	55.06
6	1	2	2	2	3	3	3	1	1	1	2	29.1	29	29	29.22	2895	2908	2939	69.29
7	1	3	3	3	1	1	1	3	3	3	2	22.9	20	19	26.33	2211	2224	2255	66.97
8	1	3	3	3	2	2	2	1	1	1	3	32.7	33	32	30.25	2844	2857	2888	69.14
9	1	3	3	3	3	3	3	2	2	2	1	33.8	35	32	30.52	3248	3251	3279	70.26
10	2	1	2	3	1	2	3	1	2	3	1	35.9	36	36	31.17	995	908	939	59.51
11	2	1	2	3	2	3	1	2	3	1	2	29.9	32	32	29.96	228	241	272	47.78
12	2	1	2	3	3	1	2	3	1	2	3	34.1	34	34	30.6	1011	1024	1055	60.25
13	2	2	3	1	1	2	3	2	3	1	3	34.2	35	35	30.8	1021	1034	1065	60.34
14	2	2	3	1	2	3	1	3	1	2	1	25.5	26	26	28.23	724	837	768	57.76
15	2	2	3	1	3	1	2	1	2	3	2	28.5	29	29	29.19	1741	1754	1785	64.91
16	2	3	1	2	1	2	3	3	1	2	2	36	38	36	31.23	1001	1014	1045	60.17
17	2	3	1	2	2	3	1	1	2	3	3	32.5	32	32	30.18	391	404	435	52.23
18	2	3	1	2	3	1	2	2	3	1	1	43.6	44	44	32.85	1141	1154	1185	61.29
19	3	1	3	2	1	3	2	1	3	2	1	33.8	34	34	30.65	221	234	265	47.53
20	3	1	3	2	2	1	3	2	1	3	2	37.5	37	38	31.46	821	834	865	58.48
21	3	1	3	2	3	2	1	3	2	1	3	35.6	34	37	31.01	451	464	495	53.42
22	3	2	1	3	1	3	2	2	1	3	3	39.8	37	38	31.67	571	584	615	55.40
23	3	2	1	3	2	1	3	3	2	1	1	45	45	42	32.84	711	724	755	57.26
24	3	2	1	3	3	2	1	1	3	2	2	36	36	35	31.04	313	326	157	46.98
25	3	3	2	1	1	3	2	3	2	1	2	33.8	33	31	30.27	1901	1914	1945	65.66
26	3	3	2	1	2	1	3	1	3	2	3	35.6	36	36	31.06	661	674	705	56.64
27	3	3	2	1	3	2	1	2	1	3	1	32.1	33	33	30.21	1771	1784	1715	64.89

## Optimization Techniques

This paper describes a grey-based Taguchi method for optimizing multi-response manufacturing problems. At first, the optimization is done with the Taguchi method for each response separately, and then subsequently, the multi-response values are dealt with grey relational analysis (GRA).

### Taguchi Method

Taguchi Method performed the optimization of performance characteristics of the FDM process. It is also primarily used to improve product quality by pursuing a methodical approach towards using a unique OA design based on the number of process parameters for conducting a small number of experiments and analyzing the experimental results. Taguchi determined the loss function as a deviation between the experimental and target values. Subsequently, the loss function expresses in terms of mean squared deviation (MSD) and, thus, signal-to-noise (S/N) ratio. Each trial of experiments had conducted three times to obtain the MSD because both measures, average and variability, are contained in the MSD. The replication determines a variance index called the signal-to-noise (S/N) ratio [24]. The S/N ratio was determined using Eq. (1) to analyze further:

1 $$S/N=-10{\mathrm{log}}_{10}\left({\mathrm{MSD}}\right),$$

where MSD is mean squared deviation from the target value of the quality characteristic.

The MSD is defined differently for each quality characteristic. In this paper, only one type of quality characteristic, "larger the better", is chosen for two responses: Tensile strength and modulus of elasticity. Eq. (2) represents the corresponding expressions for MSD for the "larger the better" type of response:

2 $$\mathrm{MSD}= \left( 1\left/ {y}_{1}^{2} \right. + 1 \left/ {y}_{2}^{2} \right.+ 1 \left/ {y}_{3}^{2}\right.+\dots +1 \left/ {y}_{n}^{2} \right. \right) \left/ n \right.,$$

where, *n* is the number of replications in each trial.

ANOVA as a standard analysis tool ascertains the significance of the factors at a 95% confidence level and their percent contributions in affecting the responses. Comparing the calculated values of the F-ratio with the tabulated value of the F-ratio at a stated significance level counts the significance or insignificance of a particular factor. A factor is said to be significant if its calculated F-ratio is greater than the tabulated F-ratio. If any, the insignificant factors pooled, and the pooled ANOVAs for the two responses, as shown in Table 3.

**Table 3:** Pooled ANOVA for tensile strength and modulus of elasticity

Ultimate tensile strength						Modulus of elasticity				
Factors	DOF	SS	V	F-ratio	P (%)	DOF	SS	V	F	P (%)
A	2	50.47	25.23	148.69	47.98	2	239.21	119.61	41.41	20.85
B	2	3.05	1.52	8.97	2.59	2	213.28	106.64	36.93	18.53
C	2	2.47	1.23	7.26	2.04	2	77.24	38.62	13.37	6.38
D	2	8.53	4.27	25.13	7.84	2	58.04	29.02	10.05	4.67
E	2	7.89	3.94	23.24	7.22	2	92.06	46.03	15.94	7.71
F	2	3.18	1.59	9.37	2.72	2	61.45	30.72	10.64	4.97
G	2	24.71	12.35	72.80	23.33	2	56.14	28.07	9.72	4.50
H	2	0.33	0.17	**Pooled**	**0.00**	2	0.06	0.03	**Pooled**	**0.00**
I	2	0.35	0.18	**Pooled**	**0.00**	2	227.17	113.59	39.33	19.78
J	2	2.49	1.24	7.33	2.06	2	71.87	35.94	12.44	5.90
K	2	0.82	0.41	**Pooled**	**0.00**	2	16.77	8.39	**Pooled**	**0.00**
Error	10	1.70	0.17		4.23	8	23.11	2.89		6.71
Total	26	104.47			100	26	1119.56			100.00

**F **_**0.05, 2, 10**_** = 4.10; F **_**0.05, 2, 8**_** = 4.45**; DOF-Degree of Freedom, SS-Sum of Squares, V-Variance, P-Percent Contribution

### Grey Relational Analysis (GRA)

Grey relational analysis (GRA) reduces the multi-response problems into a single-objective decision-making problem by merging the entire range of performance-characteristic values for every response into a single value [25]. Recent research studies have revealed that the GRA has proven substantial and powerful techniques to analyze multiple response characteristics. In the GRA, the data preprocesses into some defined quantitative indices, namely, grey relational generation, grey relational coefficients, and grey relational grade, for further analysis. In this article, a linear normalization was performed on the S/N ratio by converting an original sequence of the S/N ratio into a decimal sequence between 0.00 and 1.00 for comparison, called the grey relational generation [25, 26]. Here, Eq. (3) has used to normalize the S/N ratios:

3 $${y}_{i}^{*}(t)=\frac{{y}_{i}^{o}(t)-\mathrm{min} {y}_{i}^{o}(t)}{\mathrm{max}{ y}_{i}^{o}\left(t\right)-\mathrm{min} {y}_{i}^{o}(t)} ,$$

where ${y}_{i}^{*}(t)$ is the normalized value of *t*th performance characteristic in the *i*th experiment, ${y}_{i}^{o}(t)$ is the original sequence (S/N ratio) of *t*th performance characteristic in the *i*th experiment, and $\mathrm{max}{y}_{i}^{o}(t)$ and $\mathrm{min}{y}_{i}^{o}\left(t\right)$ are the maximum and minimum values of ${y}_{i}^{o}(t)$ respectively. The calculated grades vary between 0.00 and 1.00; if grades equal 1.00, then corresponding sequences would be identically coincident [27]. The grey relational coefficient, which establishes the relationship between the experimental (actual) value and ideal (best) value, can be determined by Eq. (4):

4 $${\varepsilon }_{i}\left(t\right)=\frac{\Delta \mathrm{min}+\delta \Delta \mathrm{max}}{{\Delta }_{oi}\left(t\right)+\delta \Delta \mathrm{max}} , 0<{\varepsilon }_{i}(t)\le 1,$$

where ${\Delta }_{oi}$ is the deviation sequence of the reference sequence $\left({y}_{o}\right)$ and the comparability sequence $\left({y}_{i}\right)$, i.e., ${\Delta }_{oi} = \Vert {y}_{o}\left(t\right)-{y}_{i}(t)\Vert$ is the absolute difference between ${y}_{o}\left(t\right)$ and ${y}_{i}(t)$, and $\delta$ is the distinguishing coefficient, and $\delta \in \left[0, 1\right]$. In the present article, the value of $\delta$ is equal to 0.5.

$\Delta \mathrm{max}={\forall }_{j}^{\mathrm{max}}\in i{\forall }_{k}^{\mathrm{max}}\Vert {y}_{o}\left(t\right)-{y}_{i}(t)\Vert$ is the maximum value of ${\Delta }_{oi}$, and $\Delta \mathrm{min}={\forall }_{j}^{\mathrm{min}}\in i{\forall }_{k}^{\mathrm{min}}\Vert {y}_{o}\left(t\right)-{y}_{i}(t)\Vert$ is the minimum value of ${\Delta }_{oi}$. Afterward, averaging the grey relational coefficient corresponding to each performance characteristic calculates the grey relation grade *ε* (*y*_*o*_, *y*_*i*_). It can be determined by Eq. (5) [26]:

5 $$\varepsilon \left({y}_{o},{y}_{i}\right)={G}_{i}=\frac{1}{q}\sum_{t=1}^{q}{\varepsilon }_{i}(t),$$

where *q* is the number of performance characteristics, in this article, the value of *q* is equal to 2. The grey relation grade expresses the similarity between comparability and original sequences.

## Analysis and Discussion of Results

### Effect of Process Parameters on Tensile Strength

**Figure 3:**
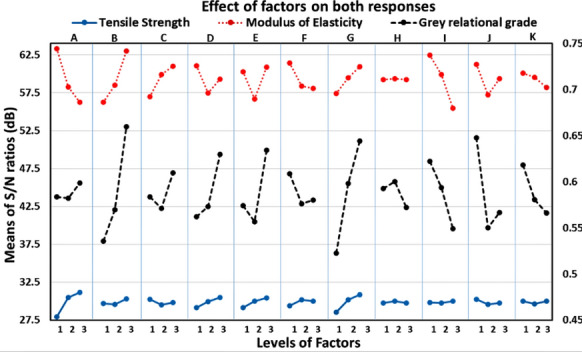
Graphical representation of effects of parameters on UTS, modulus of elasticity, and grey relational grade

It is observed from Figure 3 that tensile strength increases continuously as layer height increases. Table 3 reveals that layer height is the most influential factor contributing 47.98% to achieving the maximum tensile strength. The primary cause of the decrease in strength is the increase in the residual stresses resulting from warp deformation, volumetric shrinkage, interlayer porosity, and weak interlayer bonding [6, 28]. A high-temperature gradient will be established between the top and bottom portion of the printed part, resulting in significant thermal stresses and distortions. This phenomenon starts with 15 layers and becomes more apparent in the case of 30 layers. In this study, 13, 20, and 40 layers have formed by setting the layer height as 0.3 mm, 0.2 mm, and 0.1 mm, respectively.

The distortion effect observed due to the temperature gradient can be seen in the case of layer height selected as 0.2 mm and 0.1 mm.

**Figure 4:**
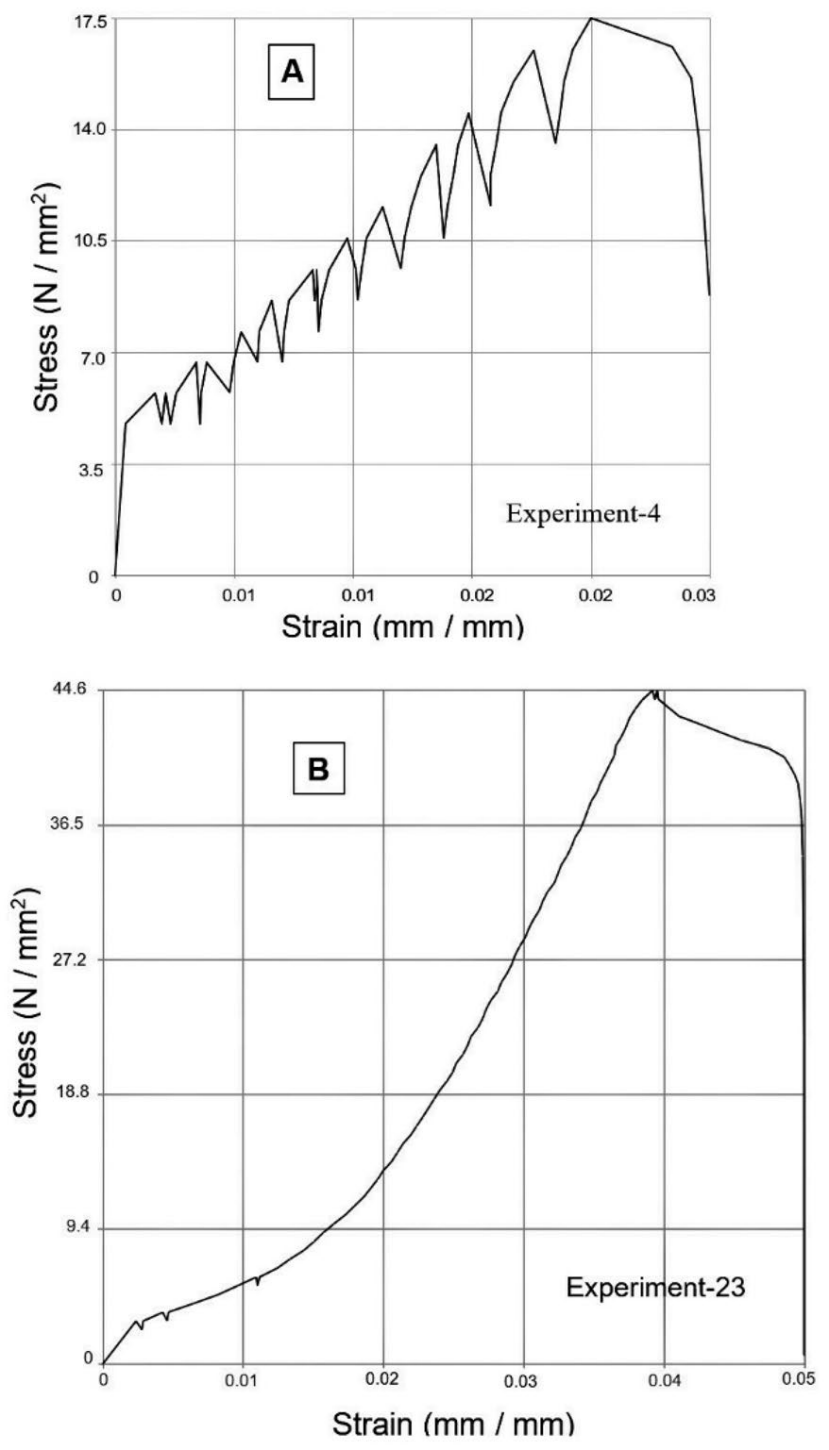
**a** Stress-strain diagram of Experiment 4 yielding minimum tensile strength, **b** stress-strain diagram of Experiment 23 yielding maximum tensile strength

As the layer height increases, fewer layers are required to print the part. Fewer layers decrease the tendency of warp deformation leading to an increase in tensile strength [6, 29, 30]. The stress-strain diagrams of specimens printed by experiment numbers 4 and 23 have been represented by Figure 4. The previous studies [3, 6, 8, 15, 30] have observed the same results.

**Figure 5:**
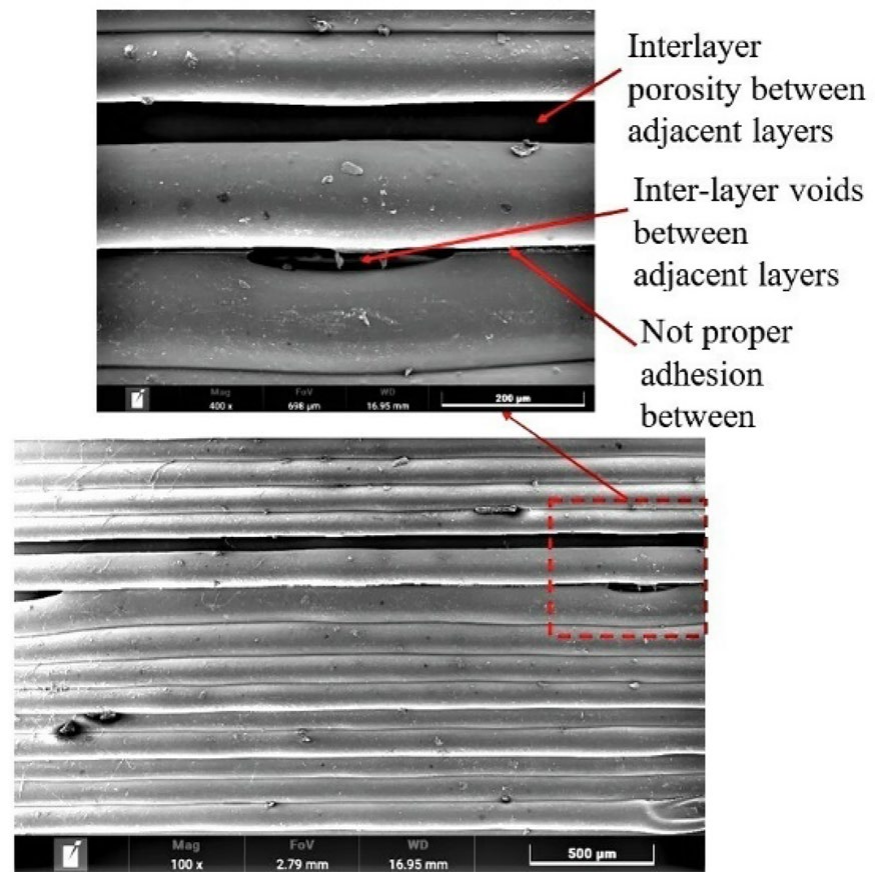
SEM micrograph of specimen obtained from Experiment 4

**Figure 6:**
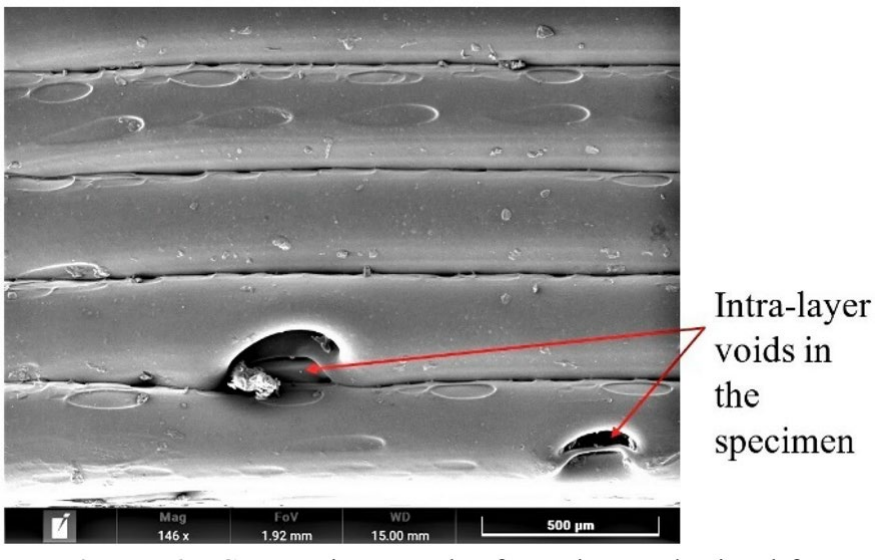
SEM micrograph of specimen obtained from Experiment 23

Scanning electron microscopy (SEM) micrograph of the specimen obtained through Experiment 4 (Figure 5) indicates that the large size of interlayer porosity and voids between the adjacent layers cause the reduction in tensile strength. On the other hand, no interlayer porosity between interlayers in the specimen observed from Experiment 23 (Figure 6), but intra-layer voids observed in the SEM may cause by already existing porosity in the filament. Thus, the specimen obtained from Experiment 23 has a higher tensile strength.

The number of shells is the second most influential factor having a percent contribution of 23.33% (Table 3) for improving tensile strength. Shell or perimeter, always deposited at the outer contour along the longitudinal direction of the component without following the path of infill pattern, will add strength irrespective of the setting of other factors. Thus, it always contributes to resisting axial tensile loading. Increasing the number of shells leads to depositing more strands imparting a more significant effect. Tensile strength increases by increasing the number of shells, as shown in Figure 3. Similar results were observed by Gordon et al. [3] and Lanzotti et al. [8].

The number of top/bottom layers is the third most influential factor for tensile strength and has a percent contribution of 7.84%. The top and bottom portions of the specimens have formed by printing the top and bottom layers of fixed line width, 0.4 mm, and a 150% flow rate for improving surface finish and strength. The printed specimen's relative density depends upon the line width of the strands. In the case of line, concentric and zig-zag patterns, choosing a 0.4 mm line width is equal to 100% relative density. The slicer software recommends a minimum of 2 top and bottom layers. If the specimen has printed with 50% infill density and 2 top and bottom layers, then voids on the top and bottom surface of it can be seen macroscopically. Thus, the number of top and bottom layers has increased to 4 to improve the surface finish and strength. Tensile strength has increased by increasing the top and bottom layers, as shown in Figure 3.

Infill density is the fourth most influential factor having a percent contribution of 7.22%. Increasing the infill density leads to forming a more solid portion in a given cross-section; hence, a dense part prints with strong intra-layer and inter-layer bonding [14]. Increasing the infill density reduces the air gap between two strands by adding a new strand. Hence, increasing the infill density increases the overall bonding degree and the tensile strength [10]. The tensile strength increases continuously with an increasing value of the infill density, as shown in Figure 3.

The flow rate has a minimal contribution of 2.72% in the tensile strength. It has already been discussed that strength improvement results from reduced inter-layer and intra-layer voids in the rasters. The intra-layer voids minimize by increasing material flow from the nozzle negotiating a turn in path [31]. The flow rate of the semi-liquid filament through a nozzle has controlled by two teethed rollers driven by a rolling step motor. With a low flow rate, cavity-type defects emerge on the component's surface because of back pressure drop in the extrusion chamber. Increasing the flow rate reduces these defects [32]. Figure 3 indicates that tensile strength continuously increases with the flow rate.

Infill pattern, print speed, and top and bottom pattern have a minor effect on tensile strength, as shown in Table 3. This study uses three kinds of infill deposition patterns and three deposition patterns of top/bottom layers. In the grid, triangular, and zig-zag deposition pattern, one completer layer has formed by depositing material through the nozzle continuously in one single path motion with frequent changes in direction, accelerating the deposition process. However, in a concentric pattern and line pattern, one complete layer is yielded by depositing the material through a set of distinct paths, resulting in retarding the deposition process. Thus, a concentric pattern coupled with a line pattern helps minimize the void formation in the intra-layers and inter-layers [31]. The nozzle runs a short distance to print one raster adjacent to another in a line pattern. Thus, the raster maintains a high temperature and tends to better fusion between adjacent rasters, responsible for the stronger bonding and good surface finish [14]. In a concentric pattern, all rasters deposit along the direction of the tensile load. Hence, the outer boundary raster firmly fuses with the shell without making any voids that will help to improve the strength. Therefore, in this study, the combination of concentric infill pattern (contour deposition method) and line pattern with 45° raster (raster deposition method) as a top/bottom surface gives the higher tensile strength with a better surface finish. Wang et al. [30] observed the same.

### Effect of Process Parameters on the Modulus of Elasticity

The ANOVA Table 3 and Figure 3 indicate the percent contribution of the significant factors and an optimal setting of the factors to achieve maximum modulus of elasticity or stiffness. The minimum value of layer height and four shells are accountable for attaining the maximum value of elastic modulus. This result is in line with the previous study [8]. Concentric infill pattern, 100% infill density, the print speed at 40 mm/s, and four shells together are responsible for increasing the tensile strength and modulus of elasticity—the reasons for it, as explained earlier. However, increasing the elastic modulus at lower layer height values, flow rate, and the number of top and bottom layers with zig-zag as the top and bottom pattern observes the opposite trend. The stress-strain diagrams obtained from specimens printed through Experiments 9 and 24, respectively, have observed the maximum and minimum values of elastic modulus.

**Figure 7:**
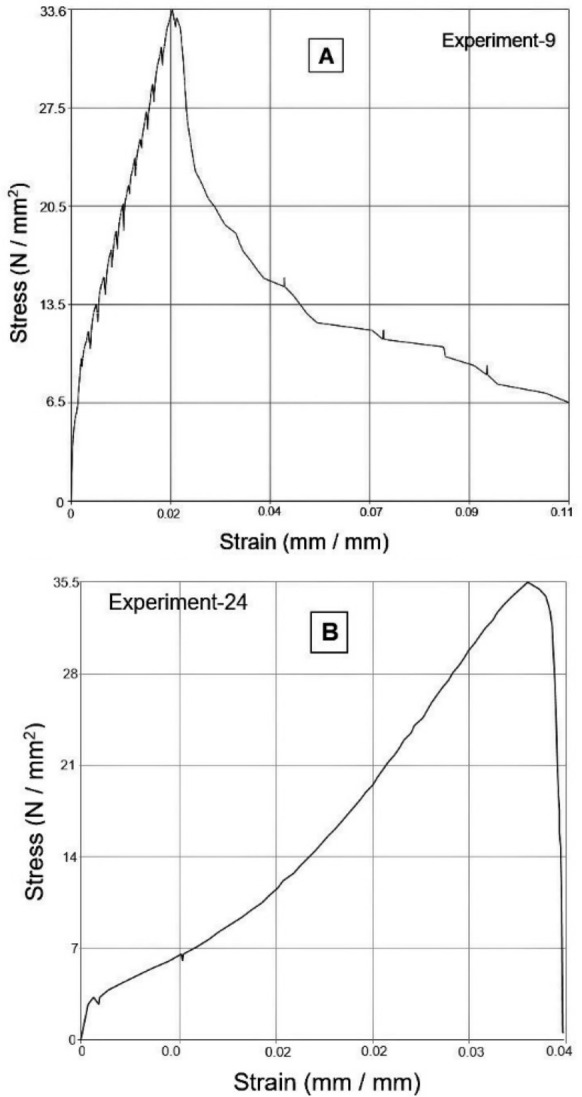
**a** Stress-strain diagram of Experiment 9, **b** stress-strain diagram of Experiment 24

In the stress-strain diagram of Experiment 9, the slope of the curve is the highest (maximum elastic modulus), as shown in Figure 7a; however, jitters observe in the strain-hardening region in the diagram. Whereas stress-strain diagram of Experiment 24, shown in Figure 7b, depicts a smaller slope (minimum elastic modulus) with a smooth curve in the strain-hardening region. It means that at 0.1 mm layer height, there is a strong cohesion in a raster and adhesion at the interface between adjacent rasters, as described in the previous study. However, at lower layer height, a large number of layers are formed; thus, a more significant thermal gradient establishes in the specimen due to the rapidly decreasing temperature of the newly deposited layer and the continuous rise of temperature at the bottom layers. Hence, considerable variation in the cooling and heating rate results in significant variation in thermal stresses and distortion in the specimen, making interface bonding somewhat brittle. The stress-strain diagram slope will be higher during such a specimen's tensile testing. However, a slip occurs when the specimen enters the plastic region and resolved shear stresses has generated at the interface. Continuously increasing load in the plastic region results in gradually fracturing these brittle-natured bonds, seen in the form of jitters in Figure 7a; the filament rasters will support the total tensile load. These rasters have strong cohesion; therefore, strain hardening indicates in Figure 7a, along with a large modulus of elasticity.

On the other hand, lower strength has obtained at 0.1 mm layer height by forming thin layers, resulting in less strength, as compared with the thick layers formed at 0.3 mm layer height having more strength. In Experiment 24 (Figure 7b), a specimen fabricated with 0.3 mm layer height gives minimum elastic modulus with sufficiently high strength. Minimum elastic modulus and high strength may be possible when cohesion and adhesion bonding is exact, i.e., ductile. Thus, in the elastic region of the stress-strain diagram, the specimen shows ductile behavior by showing a low slope of a straight line. In the plastic region, due to solid adhesion bonding, no resolved shear stresses are formed at the interface, and the specimen as a whole is elongated, showing maximum tensile strength.

Poisson’s ratio is also a remarkable mechanical property for any functional part subjected to some loading. It expresses the transverse reduction of a material due to enlarging it by applying a force in a longitudinal direction. High Poisson's ratio indicates large elastic deformation exhibited by the material even though developing a small value of strain. Poisson's ratio approaching zero means the material does not exhibit elastic deformation disregarding a finite strain value. There are various direct and indirect methods to determine Poisson's ratio. This study determines the Poisson's ratio for the tensile specimen using only uniaxial tensile testing through an extensometer. The calculated Poisson's ratio relies on longitudinal strain solely, as represented by Eq. (6) [33]. Using Taguchi’s technique for the "smaller the better" type of response, Poisson's ratio at break is around 0.20 for the prepared specimen. Poisson's ratio as a mechanical property is not investigated in this article as this study restricts measuring and analyzing the ultimate tensile strength and modulus of elasticity.

6 $$\nu =\frac{1}{\varepsilon_{l} }\left(1-{\sqrt{\frac{ V \left/ {V}_{0} \right.}{1+{\varepsilon }_{l}}}}\right) ,$$

where $\nu$ is the Poisson's ratio, ${\varepsilon }_{l}$ is the longitudinal strain, *V* is the volume at the break, ${V}_{0}$ is the original volume.

The results of grey relational analysis have reported in Table 4. In GRA, Eqs. (3)−(5) determine the grey relational generations, grey relational coefficients, and grey relational grades, respectively. The higher rank decides the higher value of the grey relational grade in Table 4. Since an accumulation of both performance characteristics obtains the grades, the maximum value of grey relational grade among 27 experiments, i.e., Experiment 9 (Table 4), exactly stands for the optimum combination of factor levels for the FDM process in this work. The analysis of variance is applied to the grey relational grades (Table 5) to ascertain the significant factors. Table 5 and Figure 3 represent the significant factors and their optimal levels viz., B_3_, D_3_, E_3_, G_3_, I_1_, J_1_, and K_1_ to integrate desired mechanical properties in the printed parts. Table 5 shows that the concentric infill pattern (B_3_) is the most influential factor, contributing 23.51%.

**Table 4:**
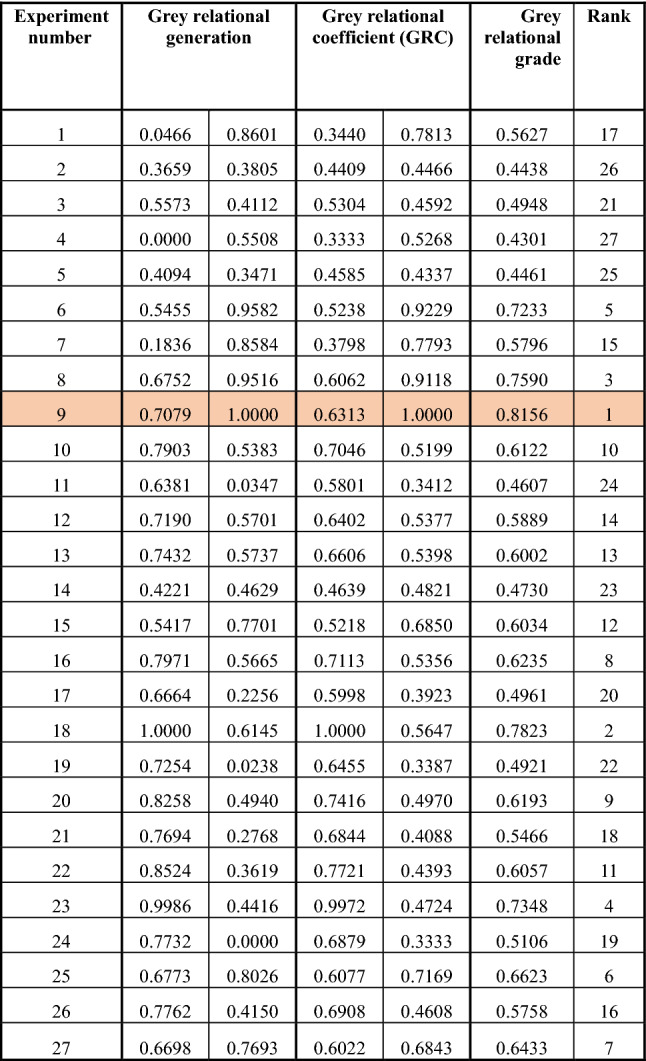
Results of grey relational analysis

**Table 5:** Pooled ANOVA for the grey relational grade (GRG)

Factors	DF	SS	V	F-ratio	P (%)
A	2	0.00152	0.00076	**Pooled**	**0.00**
B	2	0.07395	0.03698	23.33	23.51
C	2	0.00693	0.00347	**Pooled**	**0.00**
D	2	0.02362	0.01181	7.45	6.79
E	2	0.02993	0.01497	9.44	8.89
F	2	0.00558	0.00279	**Pooled**	**0.00**
G	2	0.06818	0.03409	21.51	21.59
H	2	0.00378	0.00189	**Pooled**	**0.00**
I	2	0.02436	0.01218	7.68	7.04
J	2	0.04921	0.02460	15.52	15.29
K	2	0.01279	0.00640	4.04	3.20
Error	12	0.01902	0.00159	−	13.68
Total	26	0.30104	−	−	100.00

**F **_**0.05, 2, 12**_** = 3.88**; DOF-Degree of Freedom, SS-Sum of Squares, V-Variance, P-Percent Contribution

## Confirmation Experiment

**Figure 8:**
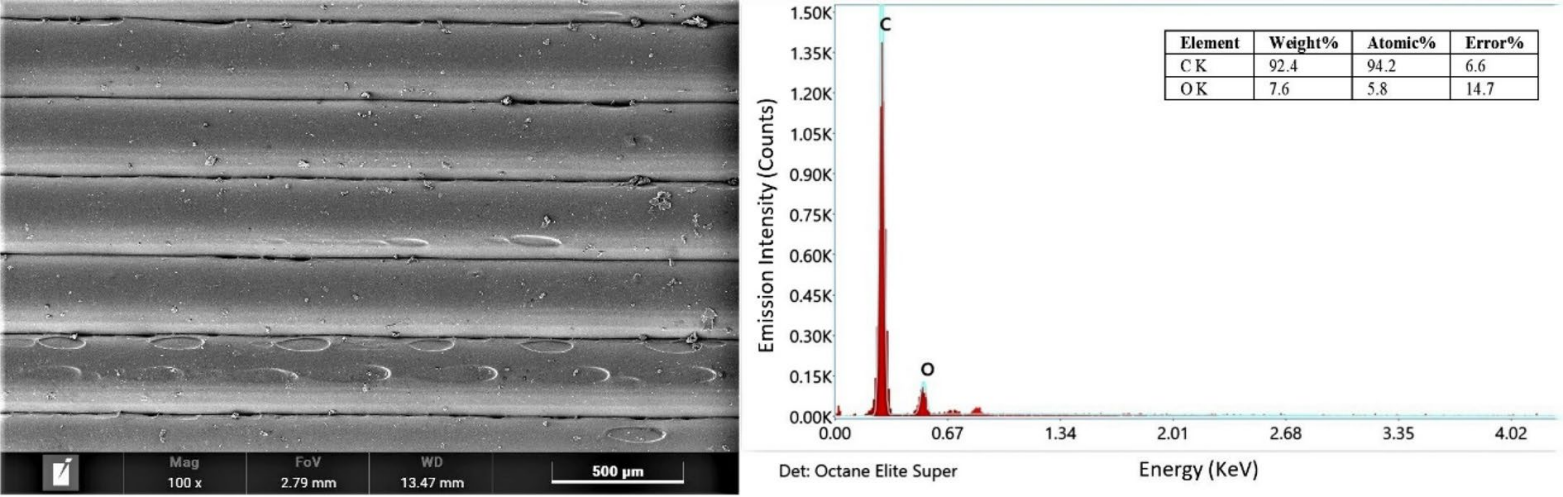
SEM micrograph of specimen obtained from confirmation experiment and EDX spectra of PLA

After choosing the optimal parametric levels, the confirmation experiment aims to verify the enhancement in the expected performance characteristics with the chosen optimal printing parameters, i.e., to verify the optimum conditions analyzed by the grey relational analysis. Experiments were conducted three times with an optimal combination of process parameters and the obtained testing results, as seen in Table 6. SEM micrograph (Figure 8) of the specimen obtained from the confirmation experiment observed the strong bonding between rasters without any defect. Therefore, the specimen has good comprehensive mechanical properties. Figure 8 also reveals the energy dispersive X-ray spectroscopy (EDX) of PLA, indicating the elements present in the specimen: 92.4 wt% carbon and 7.6 wt% oxygen. Eq. (7) [26] predicts the grey relational grade for optimal levels of the process parameters.

7 $$\widehat{\varphi }={\varphi }_{m}+\sum_{i=1}^{p}\left({\overline{\varphi } }_{i}-{\varphi }_{m}\right) ,$$

where $\widehat{\varphi }$ is the predicted grey relational grade, ${\varphi }_{m}$ is the average of grey relational grades, ${\overline{\varphi } }_{i}$ is the average value of grey relational grade at the optimal setting of the corresponding significant process parameter, and *p* is the number of significant process parameters.

$${\varphi }_{m}=0.5883,$$

thus, optimal predicted grey relational grade at optimal conditions is $\widehat{\varphi }=0.9260$.

**Table 6:** Results of confirmation experiments

	Ultimate tensile strength (MPa)	Modulus of elasticity (MPa)	Grey relational grade
Trial 1	41	3251	−
Trail 2	42	3256	−
Trail 3	42.6	3274	−
S/N ratio	36.9542	70.26	−
Initial process parameters	A_1_B_3_C_3_D_3_E_3_F_3_G_3_H_2_I_2_J_2_K_1_		0.8156
Optimal process parameters	Prediction	A_3_B_3_C_3_D_3_E_3_F_1_G_3_H_2_I_1_J_1_K_1_	0.926
	Confirmation test	A_3_B_3_C_3_D_3_E_3_F_1_G_3_H_2_I_1_J_1_K_1_	0.9524

Now, confidence interval $CI=\pm \sqrt{{F}_{\alpha }(1, n)\frac{{V}_{e}}{{N}_{e}}}$,

${F}_{\alpha }\left(1,n\right)=F;$ value at 95% confidence level for mean DOF (always=1) and DOF of error ${V}_{e}=\mathrm{Error \, Variance}=0.00159$ and $n=12$,

$${N}_{e}=\frac{\mathrm{Total \, number \, of \,results}}{\mathrm{ DOF \, of \, mean}+\mathrm{DOF \, of \, significant \, factors}},$$

$${N}_{e}=\frac{27}{1+14}=1.8,$$

$${F}_{0.05}\left(1, 12\right)=4.7472 \left(\text {tabulated} \right),$$

$$CI=\pm \sqrt{\frac{4.7472\times 0.00159}{1.8}}=\pm 0.0647,$$

$$0.9260-0.0647<\mu <0.9260+0.0647.$$

Thus, for 95% *CI*, the predicted optimum grade lies in this range: $0.8613<\mu <0.9907$.

After conducting the confirmation experiments, the value of the grey relational grade is equal to 0.9524 (Table 6), which lies within the optimal range. Thus, the grey-Taguchi multi-response optimization technique has improved the mechanical properties by up to 16.83%, as verified by the confirmation experiments.

**Figure 9:**
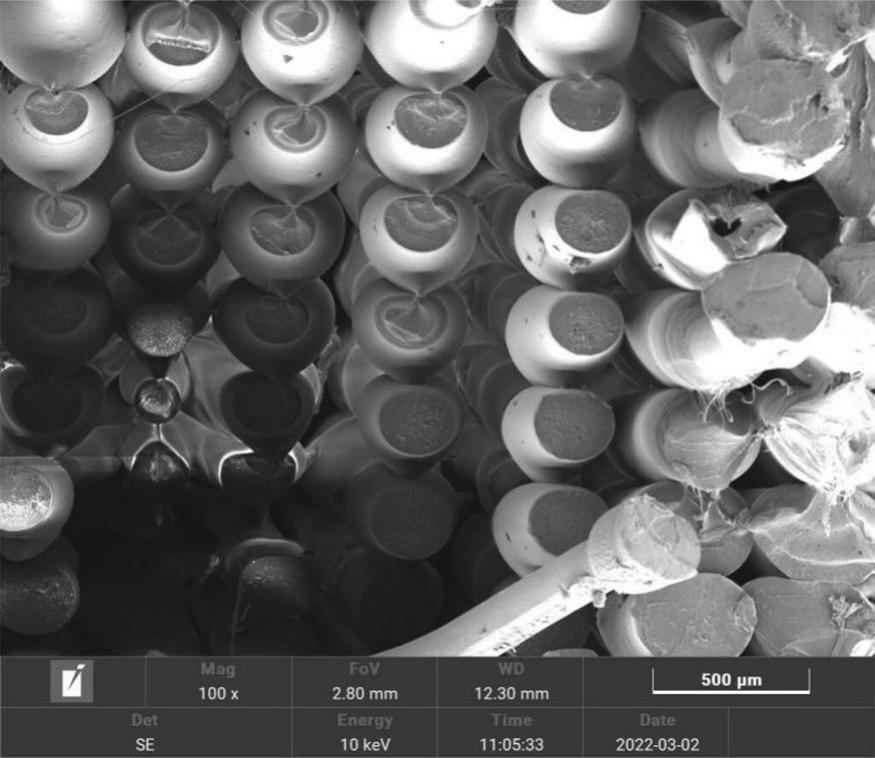
SEM micrograph of tensile fractured specimen obtained from confirmation experiment

SEM micrograph (Figure 9) of the tensile fractured specimen obtained from the confirmation experiment revealed the ductile fracture because each raster at its ends has a minimal diameter compared to its original diameter. Each raster is sufficiently elongated at the cost of reducing diameter before fracture.

## 3D Printing of Surgical Tools

**Figure 10:**
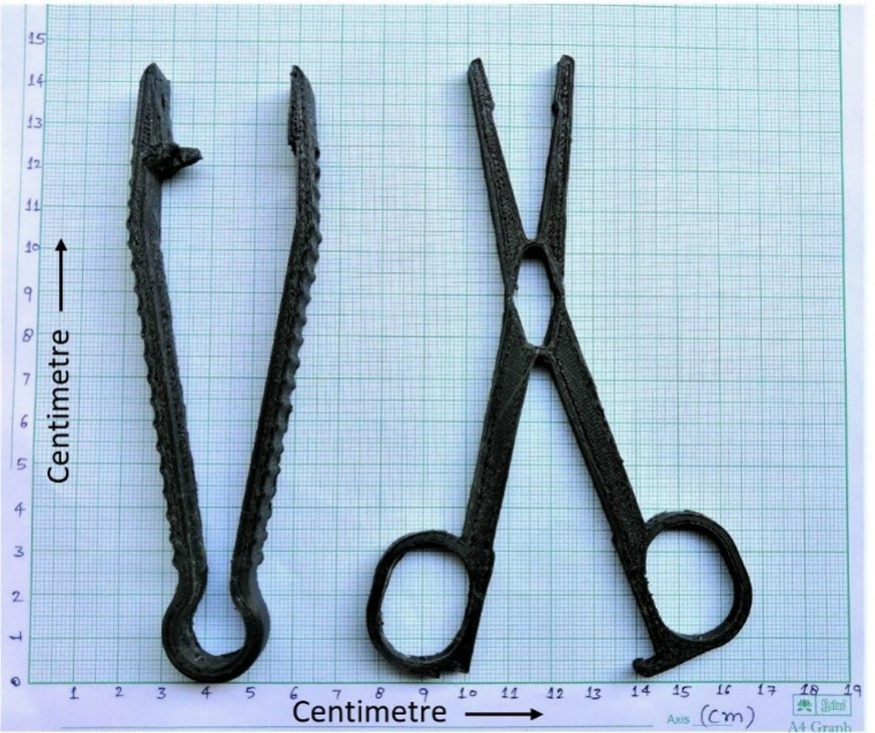
3D printed surgical tools (forceps and hemostat)

To infuse the comprehensive mechanical properties, the surgical tools (Figure 10), namely, forceps and hemostat, have been printed in horizontal orientation (*x-y* plane) using optimal levels of process parameters obtained through GRA.

## Conclusions

The prime objective of the current study was to fabricate surgical instruments. The adopted mechanical properties of PLA have unified through optimizing FDM process parameters using Taguchi method-based grey relational analysis. Based on results and discussions, the study concludes the following points:

1. The descending order of significant factors in conjunction with their levels for the sake of improving UTS are as follows: Layer height (A_3_), number of shells (G_3_), number of top/bottom layers (D_3_), infill density (E_3_), flow (F_2_), infill pattern (B_3_), print speed (J_1_) and top/bottom layer pattern (C_1_) respectively. Moreover, printing temperature (H), build plate temperature (I), and fan speed (K) observe as insignificant factors.
2. The descending order of significant factors in conjunction with their levels for the sake of enhancing modulus of elasticity are as follows: layer height (A_1_), build plate temperature (I_1_), infill pattern (B_3_), infill density (E_3_), top/bottom layer pattern (C_3_), print speed (J_1_), flow (F_3_), number of top/bottom layers (D_1_), number of shells (G_3_) respectively. Furthermore, printing temperature (H) and fan speed (K) observe as insignificant factors for modulus of elasticity.
3. The descending order of significant optimal process parameters along with their specific levels to yield comprehensive mechanical properties through GRA in the final product are as follows: infill pattern (B_3_), number of shells (G_3_), print speed (J_1_), infill density (E_3_), build plate temperature (I_1_), number of top and bottom layers (D_3_), fan speed (K_1_) respectively.
4. The effectiveness of grey relational analysis was verified by conducting a confirmation test through which the GRG of multiple performance characteristics has significantly enhanced by 16.83%.
5. Finally, the biopolymer surgical tools—forceps and hemostat—have been successfully printed through the FDM process at optimal levels of process parameters ascertained by GRA which are as under: 0.3 mm layer height, concentric infill pattern, zig-zag top/bottom pattern, four top/bottom layers, 100% infill density, 100% flow, four number of shells, 210° C printing temperature, 50° C build plate temperature, 40 mm/s printing speed, 10% capacity of fan speed. 
